# Supplementary material for: Evolution of histone 2A for chromatin compaction in eukaryotes
Source: eLife. 2014 Jun 17;3:e02792. doi: 10.7554/eLife.02792 (PMC4098067; doi:10.7554/eLife.02792)
Supplement: Supplementary file 1. — Tables of yeast strains, plasmids, and primers. DOI: http://dx.doi.org/10.7554/eLife.02792.018 [file elife02792s002.docx]

**Supplementary file 1**

Part A: List of yeast strains used in this study

| **Name** | **Mutant name** | **Description** | **Reference** |
| --- | --- | --- | --- |
| FLY142 |  | *Mat*α, *hta1-1, hta2-1, ura3-52, his3,* pFL142-*HIS3* | Lefant et al., 1996 |
| TSY107 | Parental | *Mat*a, *hta1-1*, *hta2-1*, *ura3-52*, *his3*, pJC102-*URA3* | Schuster et al., 1986 |
| FY406 | Parental | *Mat*a*, (hta1-htb1)Δ::LEU2, (hta2-htb2)Δ::TRP1, pSAB6* | Hirschhorn et al., 1995 |
| AOY001 | WT | *Mat*a, *hta1-1*, *hta2-1*, *ura3-52*, *his3*, pFL142*-HIS3* | this study |
| AOY002 | R3 | *Mat*a, *hta1-1*, *hta2-1*, *ura3-52*, *his3*, pR3-*HIS3* | this study |
| AOY004 | R11 | *Mat*a, *hta1-1*, *hta2-1*, *ura3-52*, *his3*, pR11-*HIS3* | this study |
| AOY005 | K3 | *Mat*a, *hta1-1*, *hta2-1*, *ura3-52*, *his3*, pK3-*HIS3* | this study |
| AOY009 | K20R | *Mat*a, *hta1-1*, *hta2-1*, *ura3-52*, *his3*, pK20R-*HIS3* | this study |
| AOY011 | R17K | *Mat*a, *hta1-1*, *hta2-1*, *ura3-52*, *his3*, pR17K-*HIS3* | this study |
| AOY013 | K11 | *Mat*a, *hta1-1*, *hta2-1*, *ura3-52*, *his3*, pK11-*HIS3* | this study |
| AOY014 | K3K11 | *Mat*a, *hta1-1*, *hta2-1*, *ura3-52*, *his3*, pK3K11-*HIS3* | this study |
| AOY015 | R3R11 | *Mat*a, *hta1-1*, *hta2-1*, *ura3-52*, *his3*, pR3R11-*HIS3* | this study |
| AOY020 | R6 | *Mat*a, *hta1-1*, *hta2-1*, *ura3-52*, *his3*, pR6-*HIS3* | this study |
| AOY022 | ΔGS10 | *Mat*a*, hta1-1, hta2-1, ura3-52, his3,* pΔGS10-*HIS3* | this study |
| AOY023 | R3(ΔGS10)R11 | *Mat*a*, hta1-1, hta2-1, ura3-52, his3,* pR3Δ(GS)R11-*HIS3* | this study |
| AOY024 | ΔS15 | *Mat*a*, hta1-1, hta2-1, ura3-52, his3,* pΔS15-*HIS3* | this study |
| AOY025 | R11ΔS15 | *Mat*a*, hta1-1, hta2-1, ura3-52, his3,* pR11ΔS15-*HIS3* | this study |
| AOY029 | K11ΔS15 | *Mat*a*, hta1-1, hta2-1, ura3-52, his3,* pK11ΔS15-*HIS3* | this study |
| BBY011 | WT | *Mat*a*, (hta1-htb1)Δ::LEU2, (hta2-htb2)Δ::TRP1, pJH55* | this study |
| BBY013 | ΔS15 | *Mat*a*, (hta1-htb1)Δ::LEU2, (hta2-htb2)Δ::TRP1, pΔS15* | this study |
| BBY022 | R11 | *Mat*a*, (hta1-htb1)Δ::LEU2, (hta2-htb2)Δ::TRP1, pR11* | this study |
| BBY023 | R11ΔS15 | *Mat*a*, (hta1-htb1)Δ::LEU2, (hta2-htb2)Δ::TRP1, pR11ΔS15* | this study |
| BMY003 | Parental | *Mat*a, *hta1-1*, *hta2-1*, *ura3-52*, *his3*, *Nup49-GFP(kanMX6),* pJC102-*URA3* | this study |
| BMY004 | WT | *Mat*a, *hta1-1*, *hta2-1*, *ura3-52*, *his3*, *Nup49-GFP(kanMX6),* pFL142*-HIS3* | this study |
| BMY005 | R3 | *Mat*a, *hta1-1*, *hta2-1*, *ura3-52*, *his3*, *Nup49-GFP(kanMX6),* pR3-*HIS3* | this study |
| BMY007 | R11 | *Mat*a, *hta1-1*, *hta2-1*, *ura3-52*, *his3*, *Nup49-GFP(kanMX6),* pR11-*HIS3* | this study |
| BMY008 | K3 | *Mat*a, *hta1-1*, *hta2-1*, *ura3-52*, *his3*, *Nup49-GFP(kanMX6),* pK3-*HIS3* | this study |
| BMY012 | K20R | *Mat*a, *hta1-1*, *hta2-1*, *ura3-52*, *his3*, *Nup49-GFP(kanMX6),* pK20R-*HIS3* | this study |
| BMY014 | R17K | *Mat*a, *hta1-1*, *hta2-1*, *ura3-52*, *his3*, *Nup49-GFP(kanMX6),* pR17K-*HIS3* | this study |
| BMY016 | K11 | *Mat*a, *hta1-1*, *hta2-1*, *ura3-52*, *his3*, *Nup49-GFP(kanMX6),* pK11-*HIS3* | this study |
| BMY017 | K3K11 | *Mat*a, *hta1-1*, *hta2-1*, *ura3-52*, *his3*, *Nup49-GFP(kanMX6),* pK3K11-*HIS3* | this study |
| BMY018 | R3R11 | *Mat*a, *hta1-1*, *hta2-1*, *ura3-52*, *his3*, *Nup49-GFP(kanMX6),* pR3R11-*HIS3* | this study |
| BMY038 | R6 | *Mat*a, *hta1-1*, *hta2-1*, *ura3-52*, *his3*, *Nup49-GFP(kanMX6),* pR6-*HIS3* | this study |
| BMY039 | ΔS15 | *Mat*a, *hta1-1*, *hta2-1*, *ura3-52*, *his3*, *Nup49-GFP(kanMX6),* pΔS15-*HIS3* | this study |
| BMY040 | ΔGS10 | *Mat*a, *hta1-1*, *hta2-1*, *ura3-52*, *his3*, *Nup49-GFP(kanMX6),* pΔGS10-*HIS3* | this study |
| BMY041 | R3(ΔGS10)R11 | *Mat*a, *hta1-1*, *hta2-1*, *ura3-52*, *his3*, *Nup49-GFP(kanMX6),* pR3ΔGS10R11-*HIS3* | this study |
| BMY043 | R11ΔS15 | *Mat*a, *hta1-1*, *hta2-1*, *ura3-52*, *his3*, *Nup49-GFP(kanMX6),* pR11ΔS15-*HIS3* | this study |
| BMY045 | K11ΔS15 | *Mat*a, *hta1-1*, *hta2-1*, *ura3-52*, *his3*, *Nup49-GFP(kanMX6),* pK11ΔS15-*HIS3* | this study |
| BMY501 | WT | *Mat*a, *hta1-1*, *hta2-1*, *ura3-52*, *his3*, *Pgk1-GFP(kanMX6),* pFL142*-HIS3* | this study |
| BMY502 | R11 | *Mat*a, *hta1-1*, *hta2-1*, *ura3-52*, *his3*, *Pgk1-GFP(kanMX6),* pR11-*HIS3* | this study |
| BMY503 | ΔS15 | *Mat*a*, hta1-1, hta2-1, ura3-52, his3, Pgk1-GFP(kanMX6),* pΔS15-*HIS3* | this study |
| BMY504 | R11ΔS15 | *Mat*a*, hta1-1, hta2-1, ura3-52, his3, Pgk1-GFP(kanMX6),* pR11ΔS15-*HIS3* | this study |
| BMY505 | K11 | *Mat*a, *hta1-1*, *hta2-1*, *ura3-52*, *his3*, *Pgk1-GFP(kanMX6),* pK11-*HIS3* | this study |
| BMY509 | K11ΔS15 | *Mat*a*, hta1-1, hta2-1, ura3-52, his3, Pgk1-GFP(kanMX6),* pK11ΔS15-*HIS3* | this study |
| BMY511 | WT | *Mat*a, *hta1-1*, *hta2-1*, *ura3-52*, *his3*, *Pgk1-RFP(kanMX6),* pFL142*-HIS3* | this study |
| BMY512 | R11 | *Mat*a, *hta1-1*, *hta2-1*, *ura3-52*, *his3*, *Pgk1-RFP(kanMX6),* pR11-*HIS3* | this study |
| BMY513 | ΔS15 | *Mat*a*, hta1-1, hta2-1, ura3-52, his3, Pgk1-RFP(kanMX6),* pΔS15-*HIS3* | this study |
| BMY514 | R11ΔS15 | *Mat*a*, hta1-1, hta2-1, ura3-52, his3, Pgk1-RFP(kanMX6),* pR11ΔS15-*HIS3* | this study |
| BMY515 | K11 | *Mat*a, *hta1-1*, *hta2-1*, *ura3-52*, *his3*, *Pgk1-RFP(kanMX6),* pK11-*HIS3* | this study |
| BMY519 | K11ΔS15 | *Mat*a*, hta1-1, hta2-1, ura3-52, his3, Pgk1-RFP(kanMX6),* pK11ΔS15-*HIS3* | this study |

Part B: List of yeast plasmids used in this study

| Name | Description | Reference |
| --- | --- | --- |
| pFL142 | *CEN6, ARSH4, HIS3, HTA1* | Lefant et al., 1996 |
| pR3 | *CEN6, ARSH4, HIS3, hta1-R3* | this study |
| pR11 | *CEN6, ARSH4, HIS3, hta1-R11* | this study |
| pK3 | *CEN6, ARSH4, HIS3, hta1-K3* | this study |
| pK11 | *CEN6, ARSH4, HIS3, hta1-K11* | this study |
| pK3K11 | *CEN6, ARSH4, HIS3, hta1-K3K11* | this study |
| pR3R11 | *CEN6, ARSH4, HIS3, hta1-R3R11* | this study |
| pR6 | *CEN6, ARSH4, HIS3, hta1-R6* | this study |
| pΔGS10 | *CEN6, ARSH4, HIS3, hta1-ΔG9ΔS10* | this study |
| pR3ΔGSR11 | *CEN6, ARSH4, HIS3, hta1-R3ΔG9ΔS10R11* | this study |
| pΔS15 | *CEN6, ARSH4, HIS3, hta1-ΔS15* | this study |
| pR11ΔS15 | *CEN6, ARSH4, HIS3, hta1-R11ΔS15* | this study |
| pK11ΔS15 | *CEN6, ARSH4, HIS3, hta1-K11ΔS15* | this study |
| pR3ΔS10R11ΔS15 | *CEN6, ARSH4, HIS3, hta1-R3 ΔS10R11ΔS15* | this study |

Part C: List of oligonucleotide primers used in this study

| **Name** | **Sequence** |
| --- | --- |
| R3_f | CATACATATAAAATATAAAATGTCCGGTAGAGGTAAAGGTGGTAAAGCTGGTT  CAGCTGCTAAAGC |
| R3_r | ACCGGACATTTTATATTTTATATGTATGAAATTTGTTTGTTTTGAAGTTG |
| R11_f | GTGGTAAAGCTGGTTCAGCTAGAGCTAAAGCTTCTCAATCTAG |
| R11_r | AGCTGAACCAGCTTTACCACCTTTACCACCGGAC |
| R6_f | GTCCGGTGGTAAAGGTAGAGGTAAAGCTGGTTCAGC |
| R6_r | GCTGAACCAGCTTTACCTCTACCTTTACCACCGGAC |
| K3_f | CATACATATAAAATATAAAATGTCCGGTAAAGGTAAAGGTGGTAAAGCTGGTTC |
| K11_f | GTGGTAAAGCTGGTTCAGCTAAAGCTAAAGCTTCTCAATCTAG |
| R3R11_f | CATACATATAAAATATAAAATGTCCGGTAGAGGTAAAGGTGGTAAAGCTGGTTCAGCTAGAGCTAAAGC |
| R3R11_r | AGCTGAACCAGCTTTACCACCTTTACCTC |
| K3K11_f | CATACATATAAAATATAAAATGTCCGGTAAAGGTAAAGGTGGTAAAGCTGGTTCAGCTAAAGCTAAAGCTTC |
| ΔGS10_f | gtaaaggtggtaaagctgctgctaaagcttctc |
| ΔGS10_r | gagaagctttagcagcagctttaccacctttac |
| R3ΔGS10R11_f | gtaaaggtggtaaagctgctagagctaaagcttctc |
| R3ΔGS10R11_r | gagaagctttagctctagcagctttaccacctttac |
| ΔS15_f | cagctgctaaagctcaatctagatctgc |
| ΔS15_r | gcagatctagattgagctttagcagctg |
| R11ΔS15_f | GGTAAAGCTGGTTCAGCTAGAGCTAAAGCTCAATCTAGATCTGCTAAGGC |
| R11ΔS15_r | GCCTTAGCAGATCTAGATTGAGCTTTAGCTCTAGCTGAACCAGCTTTACC |
| K11ΔS15_f | GGTAAAGCTGGTTCAGCTAAAGCTAAAGCTCAATCTAGATCTGCTAAGGC |
| K11ΔS15_r | GCCTTAGCAGATCTAGATTGAGCTTTAGCTTTAGCTGAACCAGCTTTACC |
| R3ΔS10R11ΔS15_f | gtaaaggtggtaaagctggtgctagagctaaagctcaatctagatctgctaag |
| R3ΔS10R11ΔS15_r | cttagcagatctagattgagctttagctctagcaccagctttaccacctttac |
| Probe2_P1_f | CTCGTCCTCTGAACCAAAGC |
| Probe2_P1_r | ACCACATCGTTGTCCTCACA |
| Probe2_P2_f | TGACGAGCCATCTTTGTCAG |
| Probe2_P2_r | CCAGTAGGCGGTTGAATGTT |
| Probe2_P3_f | AGTGGAAACCACCGTTTCTG |
| Probe2_P3_r | CACTAGCGGCAGTTGATTGA |
| H2A_dR3_f | gagatatacatatgtcaggaggcaaacaaggcgg |
| H2A_dR3_r | ccgccttgtttgcctcctgacatatgtatatctc |
| H2A_dR11_f | acaaggcggtaaaaccgctaaggccaagactc |
| H2A_dR11_r | gagtcttggccttagcggttttaccgccttgt |
| H2A_R11A_f | caaggcggtaaaaccgccgctaaggccaagac |
| H2A_R11A_r | gtcttggccttagcggcggttttaccgccttg |
| H2A_R11K_f | caaacaaggcggtaaaaccaaggctaaggccaagactcgct |
| H2A_R11K_r | agcgagtcttggccttagccttggttttaccgccttgtttg |
| H2A_Δ1-12_f | CCGAGGAGATCTGCCGCCGCGATCGCCATGAAGGCCAAGTCGCGCTCGTCCCGCGCTGGCCT |
| H2A_Δ1-12_r | AGGCCAGCGCGGGACGAGCGCGACTTGGCCTTCATGGCGATCGCGGCGGCAGATCTCCTCGG |
| F_H2A_wt_EcoRI | GGCCCGAATTCTCTCAGGAAGAGGCAAACAAGGCGG |
| F_H2AdelR3_EcoRI | GGCCCGAATTCTCTCAGGAGGCAAACAAGGCGG |
| R_H2A_NotI | TTACAGTCTGCGGCCGCCTATCACTTGCTCTTGGCCGACTTG |
