# Supplementary material for: Evolution of histone 2A for chromatin compaction in eukaryotes
Source: eLife. 2014 Jun 17;3:e02792. doi: 10.7554/eLife.02792 (PMC4098067; doi:10.7554/eLife.02792)
Supplement: Supplementary file 2. — Table of yeast FISH results. DOI: http://dx.doi.org/10.7554/eLife.02792.019 [file elife02792s003.docx]

**Supplementary file 2**

Yeast FISH Data

| **Yeast FISH - Probe Set A** | | | | | | | |
| --- | --- | --- | --- | --- | --- | --- | --- |
| **Strain** | **nm** | | | | **% change** | **p-value** | **No. cells** |
|  | **Minimum** | **Maximum** | **Mean** | **Median** |  |  |  |
| WT | 364 | 1487 | 757 | 714 | 0 | 1.0E+00 | 63 |
| R3 | 178 | 1377 | 619 | 584 | -18 | 9.5E-04 | 90 |
| R11 | 22 | 1101 | 584 | 605 | -23 | 8.6E-04 | 49 |
| R3R11 | 79 | 1102 | 557 | 553 | -26 | 8.2E-06 | 72 |
| R3(ΔGS10)R11 | 107 | 1410 | 516 | 497 | -32 | 2.1E-06 | 51 |
| R11ΔS15 | 67 | 1378 | 455 | 415 | -40 | 3.9E-08 | 57 |
| K3 | 272 | 1378 | 749 | 772 | -1 | 8.6E-01 | 64 |
| K11 | 236 | 1421 | 808 | 820 | 7 | 3.1E-01 | 56 |
| K3K11 | 164 | 1430 | 747 | 751 | -1 | 8.3E-01 | 71 |
| K11ΔS15 | 124 | 1355 | 665 | 659 | -12 | 9.2E-02 | 42 |
| ΔGS10 | 66 | 1084 | 649 | 666 | -14 | 3.2E-02 | 60 |
| ΔS15 | 30 | 1236 | 668 | 726 | -12 | 9.4E-02 | 49 |
| R6 | 106 | 1410 | 674 | 672 | -11 | 5.6E-02 | 73 |
| K20R | 205 | 1539 | 708 | 686 | -6 | 3.0E-01 | 64 |
| R17K | 230 | 1598 | 743 | 699 | -2 | 7.9E-01 | 54 |
| **Yeast FISH - Probe Set B** | | | | | | | |
| WT | 259 | 1372 | 626 | 599 | 0 | 1.0E+00 | 59 |
| R11 | 64 | 1231 | 514 | 476 | -18 | 1.4E-02 | 60 |
| ΔS15 | 88 | 1452 | 586 | 549 | -6 | 2.8E-01 | 53 |
| R11ΔS15 | 66 | 934 | 446 | 433 | -29 | 2.3E-05 | 51 |
| **Yeast FISH - Probe Set C** | | | | | | | |
| WT | 91 | 1192 | 493 | 485 | 0 | 1.0E+00 | 75 |
| R11 | 74 | 1481 | 399 | 349 | -19 | 1.4E-03 | 50 |
| ΔS15 | 153 | 896 | 456 | 437 | -8 | 1.9E-01 | 55 |
| R11ΔS15 | 89 | 925 | 398 | 410 | -19 | 5.4E-03 | 48 |
| **Yeast FISH - Probe Set D** | | | | | | | |
| WT | 65 | 1029 | 361 | 342 | 0 | 1.0E+00 | 85 |
| R11 | 41 | 802 | 299 | 261 | -17 | 4.8E-05 | 70 |
| ΔS15 | 33 | 648 | 323 | 324 | -11 | 8.7E-02 | 51 |
| R11ΔS15 | 16 | 748 | 273 | 237 | -24 | 1.6E-07 | 62 |
| **Yeast (FY406) FISH - Probe Set A** | | | | | | | |
| WT | 172 | 1318 | 728 | 723 | 0 | 1.0E+00 | 80 |
| R11 | 56 | 1158 | 609 | 617 | -16 | 7.5E-03 | 86 |
| ΔS15 | 165 | 1379 | 730 | 766 | 0 | 9.7E-01 | 62 |
| R11ΔS15 | 23 | 1225 | 532 | 512 | -27 | 8.5E-05 | 61 |
