# Supplementary material for: Evolution of histone 2A for chromatin compaction in eukaryotes
Source: eLife. 2014 Jun 17;3:e02792. doi: 10.7554/eLife.02792 (PMC4098067; doi:10.7554/eLife.02792)
Supplement: Supplementary file 3. — Table of yeast nuclear and cellular volumes. DOI: http://dx.doi.org/10.7554/eLife.02792.020 [file elife02792s004.docx]

**Supplementary file 3**

Yeast Nuclear Volume Data

| **Yeast nuclear volume (TSY107 Background)** | | | | | | | |
| --- | --- | --- | --- | --- | --- | --- | --- |
| **Nuclear Vol** | **µm^3^** | | | | **% change** | **p-value** | **No. cells** |
|  | **Minimum** | **Maximum** | **Mean** | **Median** |  |  |  |
| WT | 0.6 | 6.9 | 2.9 | 2.8 | 0 | 1.0E+00 | 188 |
| R3 | 0.6 | 6.5 | 2.8 | 2.6 | -5 | 4.1E-01 | 177 |
| R11 | 0.7 | 7.0 | 2.3 | 2.1 | -20 | 5.9E-05 | 201 |
| R3R11 | 0.4 | 6.9 | 2.5 | 2.3 | -16 | 3.0E-03 | 180 |
| R3(ΔGS10)R11 | 0.7 | 8.1 | 3.1 | 3.0 | 6 | 3.7E-01 | 196 |
| R11ΔS15 | 0.8 | 6.2 | 2.8 | 2.7 | 0 | 4.2E-01 | 150 |
| K3 | 0.5 | 9.1 | 3.3 | 3.1 | 13 | 9.4E-03 | 181 |
| K11 | 0.7 | 10.8 | 3.2 | 2.9 | 3 | 2.6E-01 | 191 |
| K3K11 | 1.0 | 9.3 | 3.8 | 3.6 | 31 | 5.4E-08 | 172 |
| K11ΔS15 | 0.8 | 8.9 | 3.0 | 2.9 | 2 | 6.6E-01 | 186 |
| ΔGS10 | 0.8 | 12.6 | 3.5 | 3.1 | 10 | 3.0E-02 | 198 |
| ΔS15 | 0.8 | 8.8 | 3.3 | 3.0 | 9 | 9.7E-03 | 202 |
| R6 | 0.8 | 10.3 | 3.4 | 3.1 | 10 | 1.0E-03 | 201 |
| K20R | 0.7 | 9.3 | 3.3 | 3.0 | 7 | 1.5E-02 | 199 |
| R17K | 0.7 | 8.3 | 3.0 | 2.5 | 0 | 2.7E-01 | 199 |
| **Yeast nuclear volume (FY406 Background)** | | | | | | | |
| WT | 1.0 | 5.1 | 2.5 | 2.3 | 0 | 1.0E+00 | 268 |
| R11 | 1.0 | 3.9 | 2.0 | 1.9 | -17 | 7.0E-07 | 195 |
| ΔS15 | 1.0 | 5.0 | 2.4 | 2.1 | -3 | 4.9E-01 | 194 |
| R11ΔS15 | 1.0 | 4.2 | 2.0 | 1.8 | -19 | 1.9E-07 | 191 |
| **Yeast cellular volume (TSY107 Background)** | | | | | | | |
| WT | 11.4 | 92.8 | 46.1 | 42.6 | 0 | 1.0E+00 | 178 |
| R11 | 12.1 | 155.9 | 48.2 | 43.4 | 4 | 2.9E-01 | 199 |
| K11 | 10.7 | 114.1 | 44.2 | 44.2 | -4 | 5.0E-01 | 209 |
| ΔS15 | 13.7 | 123.5 | 47.0 | 47.0 | 2 | 5.0E-01 | 83 |
| R11ΔS15 | 13.0 | 99.5 | 46.0 | 46.0 | 0 | 4.3E-01 | 104 |
