# Supplementary material for: Evolution of histone 2A for chromatin compaction in eukaryotes
Source: eLife. 2014 Jun 17;3:e02792. doi: 10.7554/eLife.02792 (PMC4098067; doi:10.7554/eLife.02792)
Supplement: Supplementary file 4. — Table of human FISH results. DOI: http://dx.doi.org/10.7554/eLife.02792.021 [file elife02792s005.docx]

**Supplementary file 4**

Human FISH Data

| **Human FISH – IMR90 cells – HA Tag** | | | | | | | |
| --- | --- | --- | --- | --- | --- | --- | --- |
| **IMR90** | **nm** | | | | **% change** | **p-value** | **No. cells** |
|  | **Minimum** | **Maximum** | **Mean** | **Median** |  |  |  |
| WT | 35 | 1079 | 312 | 293 | 0 | 1.0E+00 | 94 |
| ΔR3 | 57 | 702 | 363 | 406 | 16 | 8.3E-03 | 53 |
| R11K | 90 | 975 | 382 | 351 | 23 | 2.3E-02 | 40 |
| R11A | 28 | 1087 | 437 | 420 | 40 | 1.05E-05 | 62 |
| ΔR3R11A | 70 | 1161 | 420 | 394 | 43 | 3.5E-03 | 54 |
| R11H | 71 | 892 | 399 | 325 | 28 | 1.1E-02 | 52 |
| R11C | 84 | 1138 | 417 | 389 | 34 | 4.4E-04 | 53 |
| R11P | 74 | 1047 | 485 | 460 | 56 | 5.9E-06 | 50 |
| **Human FISH – IMR90 cells – FLAG Tag** | | | | | | | |
| WT | 53 | 1080 | 354 | 324 | 0 | 1.0E+00 | 40 |
| Δ1-12 | 148 | 977 | 481 | 478 | 36 | 4.9E-03 | 39 |
| **Human FISH – MDA-MB-453 cells** | | | | | | | |
| WT | 33 | 745 | 296 | 293 | 0 | 1.0E+00 | 49 |
| ΔR3 | 42 | 740 | 341 | 315 | 15 | 1.9E-01 | 45 |
| R11A | 97 | 793 | 362 | 338 | 22 | 1.7E-02 | 60 |
| ΔR3R11A | 46 | 764 | 388 | 399 | 31 | 5.9E-03 | 35 |
