# Supplementary material for: Evolution of histone 2A for chromatin compaction in eukaryotes
Source: eLife. 2014 Jun 17;3:e02792. doi: 10.7554/eLife.02792 (PMC4098067; doi:10.7554/eLife.02792)
Supplement: Supplementary file 5. — Table of human nuclear area results. DOI: http://dx.doi.org/10.7554/eLife.02792.022 [file elife02792s006.docx]

**Supplementary file 5**

Human Nuclear Area Data

| **Human nuclear area - IMR90 cells - HA Tag** | | | | | | |
| --- | --- | --- | --- | --- | --- | --- |
| **IMR90** | **µm^2^** | | | **% change** | **p-value** | **No. cells** |
|  | **Minimum** | **Maximum** | **Mean** |  |  |  |
| WT | 52 | 179 | 99 | 0 | 1.0E+00 | 93 |
| ΔR3 | 73 | 372 | 142 | 43 | 1.3E-08 | 42 |
| R11K | 69 | 182 | 113 | 14 | 1.2E-03 | 64 |
| R11A | 64 | 239 | 121 | 22 | 5.6E-07 | 121 |
| ΔR3R11A | 63 | 379 | 133 | 34 | 5.9E-04 | 40 |
| R11H | 68 | 184 | 104 | 5 | 6.8E-02 | 67 |
| R11C | 53 | 153 | 98 | -1 | 4.1E-01 | 67 |
| R11P | 50 | 220 | 105 | 6 | 3.4E-01 | 67 |
| **Human nuclear area - IMR90 cells - FLAG Tag** | | | | | | |
| WT | 63 | 223 | 114 | 0 | 1.0E+00 | 50 |
| Δ1-12 | 92 | 240 | 131 | 15 | 2.8E-04 | 57 |
| **Human nuclear area - HEK293 cells** | | | | | | |
| WT | 58 | 158 | 96 | 0 | 1.0E+00 | 52 |
| ΔR3 | 65 | 161 | 106 | 10 | 7.0E-03 | 60 |
| R11A | 68 | 231 | 116 | 21 | 4.4E-05 | 65 |
| ΔR3R11A | 76 | 251 | 121 | 26 | 4.6E-07 | 52 |
| **Human nuclear area - MDA-MB-453 cells** | | | | | | |
| WT | 57 | 137 | 92 | 0 | 1.0E+00 | 36 |
| ΔR3 | 67 | 165 | 103 | 12 | 3.0E-02 | 34 |
| R11A | 92 | 172 | 132 | 43 | 4.8E-11 | 33 |
| ΔR3R11A | 78 | 154 | 111 | 21 | 3.1E-05 | 33 |
